# Site-specific dose-response relationships for cancer induction from the combined Japanese A-bomb and Hodgkin cohorts for doses relevant to radiotherapy

**DOI:** 10.1186/1742-4682-8-27

**Published:** 2011-07-26

**Authors:** Uwe Schneider, Marcin Sumila, Judith Robotka

**Affiliations:** 1 Radiotherapy Hirslanden AG, Institute for Radiotherapy, Rain 34, 5001 Aarau, Switzerland; 2 Vetsuisse Facutly, University of Zürich, Winterthurerstrasse 260, 8057 Zürich, Switzerland

## Abstract

**Background and Purpose:**

Most information on the dose-response of radiation-induced cancer is derived from data on the A-bomb survivors. Since, for radiation protection purposes, the dose span of main interest is between zero and one Gy, the analysis of the A-bomb survivors is usually focused on this range. However, estimates of cancer risk for doses larger than one Gy are becoming more important for radiotherapy patients. Therefore in this work, emphasis is placed on doses relevant for radiotherapy with respect to radiation induced solid cancer.

**Materials and methods:**

For various organs and tissues the analysis of cancer induction was extended by an attempted combination of the linear-no-threshold model from the A-bomb survivors in the low dose range and the cancer risk data of patients receiving radiotherapy for Hodgkin's disease in the high dose range. The data were fitted using organ equivalent dose (OED) calculated for a group of different dose-response models including a linear model, a model including fractionation, a bell-shaped model and a plateau-dose-response relationship.

**Results:**

The quality of the applied fits shows that the linear model fits best colon, cervix and skin. All other organs are best fitted by the model including fractionation indicating that the repopulation/repair ability of tissue is neither 0 nor 100% but somewhere in between. Bone and soft tissue sarcoma were fitted well by all the models. In the low dose range beyond 1 Gy sarcoma risk is negligible. For increasing dose, sarcoma risk increases rapidly and reaches a plateau at around 30 Gy.

**Conclusions:**

In this work OED for various organs was calculated for a linear, a bell-shaped, a plateau and a mixture between a bell-shaped and plateau dose-response relationship for typical treatment plans of Hodgkin's disease patients. The model parameters (α and R) were obtained by a fit of the dose-response relationships to these OED data and to the A-bomb survivors. For any three-dimensional inhomogenous dose distribution, cancer risk can be compared by computing OED using the coefficients obtained in this work.

## Introduction

The dose-response relationship for radiation carcinogenesis up to one or two Gy has been quantified in several major analyses of the atomic bomb survivors data. Recent papers have been published, for example, by Preston *et al. *[1,2] and Walsh *et al. *[3,4]. This dose range is important for radiation protection purposes where low doses are of particular interest. However, it is also important to know the shape of the dose-response curve for radiation induced cancer for doses larger than one Gy. In patients who receive radiotherapy, parts of the patient volume can receive high doses and it is therefore of great importance to know the risk for the patient to develop a cancer which could have been caused by the radiation treatment.

There is currently much debate concerning the shape of the dose-response curve for radiation-induced cancer [5-17]. It is not known whether cancer risk as a function of dose continues to be linear or decreases at high dose due to cell killing or levels off due to, for example, a balance between cell killing and repopulation effects. The work presented here, aims to clarify the dose-response shape for the radiotherapy dose range. In this dose range, the linear-no-threshold model (LNT) derived from the atomic bomb survivors from Hiroshima and Nagasaki can be combined with cancer risk data available from about 30,000 patients with Hodgkin's disease who were irradiated with localized doses of up to around 40 Gy.

The usual method for obtaining empirical dose-response relationships for radiation associated cancer is to perform a case control study. For each patient with a second cancer the location of, and the point dose at the malignancy can be determined. If the dose is obtained also for a number of controls the dose-response relationship for radiation induced cancer can be obtained. The advantage of this method is a direct determination of risk as a function of point dose, the major disadvantage are the large errors involved when determining the location and dose to the origin of the tumor. In this work another method was used by assuming certain shapes of dose-response curves based on model assumptions. The free model parameters for each organ are adjusted in two steps. First, the models have to reproduce in the limit of low dose the risk coefficients of the A-bomb survivors. Second, by applying the models to typical dose-volume histograms of treated patients they have to predict the corresponding observed second cancer risk which was obtained from epidemiological studies. An advantage of this method is that no point dose estimates at the tumor origin are necessary; a disadvantage is that the obtained dose-response curve is dependent on the a priori model.

The aim of this paper is to attempt a combination of the LNT model derived from the atomic bomb survivors and cancer risk data from a Hodgkin cohort treated with radiotherapy, in order to determine possible dose-response relationships for radiation associated site specific solid cancers for radiotherapy doses. This work is an extension of recently published results on possible dose-response relationships for radiation induced solid cancers for all organs combined [11,14,18]. The main difference to previous work is the use of a more realistic dose-response relationship including fractionation effects which is more suitable for radiotherapy applications. Many problems and uncertainties are involved in combing these two data-sets. However, since very little is currently known about the shape of dose-response relationships for radiation-induced cancer in the radiotherapy dose range, this approach could be regarded as an attempt to acquire more information in this area.

## Materials and methods

### Cancer risk from the Atomic bomb survivor data

The excess absolute risk in a small volume element of an organ (*EAR*) is factorized into a function of dose *RED(D) *and a modifying function that depends on the variables age at exposure (*agex*) and age attained (*agea*):(1)

where *RED *(risk equivalent dose) is the dose-response relationship for radiation induced cancer in units of dose and *β *is the initial slope, which is the slope of the dose-response curve at low dose. The modifying function *μ *contains the population dependent variables:(2)

In this form the fit parameters are gender-averaged and centered at an age at exposure of 30 years and an attained age of 70 years. The initial slopes *β*_*EAR *_and the age modifying parameters *γ*_*e *_and *γ*_*a *_for a Japanese population and for different sites are taken from Preston *et al. *[1] and are listed in Table 1.

**Table 1:** Initial slopes *β *(in brackets 95% confidence interval) of the A-bomb survivors for age at exposure of 30 and attained age of 70 years and age modifying parameters *γ*_*e *_and *γ*_*a *_for different sites.

Site	***β***_***EAR ***_- Japan*	***β***_***EAR ***_- UK*	***γ***_***e***_	***γ***_***a***_
All solid	52 (43...60)	74 (61...86)	-0.024	2.38
Female breast	9.2 (6.8...12)	8.2 (6.1...11)	-0.037	1.7
Lung	7.5 (5.1...10)	8.0 (5.5...11)	0.002	4.23
Rectum	0.56 (-0.13...1.4)	0.73 (-0.17...1.8)	-0.024	2.38
Colon	8.0 (4.4...12)	7.4 (4.0...11.0)	-0.056	6.9
Mouth and pharynx^◇^	0.56 (0.2...1.2)	0.73 (0.3...1.6)	-0.024^‡^	2.38^‡^
Esophagus	0.58 (0.18...1.1)	3.2 (1.0...6.1)	-0.002+	1.9+
Stomach	9.5 (6.1...14)	5.2 (3.4...7.7)	-0.002	1.9
Small Intestine^†^	8.0 (4.4...12)	10 (5.7...16)	-0.056	6.9
Liver	4.3 (0.0...7.2)	2.4 (0.0...4.0)	-0.021	3.6
Cervix	0.56 (0.0...1.9)	0.73 (0.0...2.5)	-0.024^‡^	2.38^‡^
Bladder	3.2 (1.1...5.4)	3.8 (1.3...6.5)	-0.024^‡^	2.38^‡^
Skin	0.35 (0.03...1.1)	0.46 (0.04...1.43)	-0.61	4.36
Brain and CNS	0.51 (0.17...0.95)	0.70 (0.23...1.31)	-0.024^‡^	2.38^‡^
Thyroid	1.2 (0.5...2.2)	0.40 (0.2...0.8)	-0.046	0.6
Salivary Gland^◇^	0.56 (0.2...1.2)	0.73 (0.26...1.6)	-0.024^‡^	2.38^‡^
Bone	-	-	-0.013^‡^	-0.56^‡^
Soft tissue	-	-	-0.013^‡^	-0.56^‡^

The values for the Japanese population (*β*_*EAR *_*- Japan*) were taken from the analysis of Preston *et al. *[1]. Risk was transferred to a Western population (*β*_*EAR *_*- UK*) by establishing a ERR-EAR weighting according to ICRP 103 [19].

*excess cases per 10'000 PY Gy, †initial slope from colon, ^◇ ^initial slope from oral cavity, +age dependence from stomach, ^‡^age dependence from all solid

In this work it is intended to combine the Japanese A-bomb survivor data with secondary cancer data from of Hodgkin's patients from a Western population. This raises the issue of risk transfer between Japanese and Western populations. In this work we transfer risk according to ICRP 103 [19] by establishing a weighting of ERR (excess relative risk) and EAR that provides a reasonable basis for generalizing across populations with different baseline risks. For this purpose *ERR:EAR *weights of 0:100% were assigned for breast, 100:0% for thyroid and skin, 30:70% for lung, and 50:50% for all others [19]. The risk ratios *ERR:EAR *from [20] are listed in Table 2 for the Japanese and UK population normalized to the initial slopes *β*_*EAR *_of a Japanese population. The ratio of the *ERR:EAR *weighted initial slope for a UK population and a Japanese population is given in the last column of Table 2. This ratio was used to transfer *β*_*EAR *_of the Japanese population to a UK population listed in the second column of Table 1.

**Table 2:** Transfer of risks between the Japanese and the UK population using weighting between a generalized ERR and EAR model according to ICRP 103 [19] and UNSCEAR [20].

Site	***β***_***ERR***_***/β***_***EAR ***_***- Japan***	***β***_***ERR***_***/β***_***EAR ***_***- UK***	***w***_***ERR***_***/w***_***EAR***_	***β***_***weighted ***_***- UK/β***_***EAR ***_***- Japan***
All solid	1.14/1.00	1.95/0.91	0.5/0.5	1.43
Female breast	1.04/1.00	2.88/0.89	0/1	0.89
Lung	1.46/1.00	1.56/0.87	0.3/0.7	1.07
Rectum^†^	1.01/1.00	1.70/0.90	0.5/0.5	1.30
Colon	0.96/1.00	0.92/0.93	0.5/0.5	0.92
Mouth and pharynx^†^	1.01/1.00	1.70/0.90	0.5/0.5	1.30
Esophagus	8.20/1.00	10.0/1.00	0.5/0.5	5.50
Stomach	0.69/1.00	0.13/0.97	0.5/0.5	0.55
Small Intestine^†^	1.01/1.00	1.70/0.90	0.5/0.5	1.30
Liver	2.03/1.00	0.19/0.93	0.5/0.5	0.56
Cervix^†^	1.01/1.00	1.70/0.90	0.5/0.5	1.30
Bladder 1	0.95/1.00	1.59/0.82	0.5/0.5	1.20
Bladder 2	0.95/1.00	1.59/0.82	0.5/0.5	1.20
Skin^†^	1.01/1.00	1.70/0.90	1/0	1.30
Brain and CNS	0.94/1.00	1.76/1.00	0.5/0.5	1.38
Thyroid	0.45/1.00	0.35/0.98	1/0	0.35
Salivary Gland^†^	1.01/1.00	1.70/0.90	0.5/0.5	1.30
Bone	39.0/1.00	48.0/1.00	0/1	1.00
Soft tissue^†^	1.01/1.00	1.70/0.90	0.5/0.5	1.30

All values are normalized to EAR of the Japanese population. The weighting *w *between the model is taken from ICRP103 [19].

^†^transfer between populations and models taken from "all other solids" of UNSCEAR [20]

### Application of cancer risk models to radiotherapy patients

A word of caution is necessary here. *EAR *as defined by Eq. 1 is the mathematically modeled excess absolute risk in a small volume element of an organ or tissue and must be distinguished from the usually used epidemiologically obtained excess absolute risk for a whole organ *EAR*^*org*^. Although this notation might appear confusing we followed this approach as it was previously used by other authors [16,17]. If the dose-volume-histogram *V(d) *in an organ of interest is known, excess absolute risk in that organ can be obtained with Eq. 1 by a convolution of the dose-volume histogram with *EAR*:(3)

where *V*_*T *_is the total organ volume and the sum is taken over all bins of the dose-volume histogram *V(D)*. For a completely homogenously irradiated organ with a dose *D*_*hom *_excess absolute risk is simply *EAR*^*org *^*= EAR(D*_*hom*_*).*

If risk estimates are applied to radiotherapy patients it is usually of interest to know the advantage of a treatment plan A relative to another treatment plan B with respect to cancer induction in one organ and one patient (same gender, age at exposure and age attained). It is therefore necessary to evaluate the risk ratio:(4)

where we introduced organ equivalent dose (*OED*) [11] which is a dose-response (*RED*) weighted dose variable averaged over the whole organ volume:(5)

It becomes instantly clear that risk ratios for different radiotherapy treatment plans are equivalent to *OED *ratios which can be simply determined on the basis of an organ specific dose-response relationship (*RED*) and dose volume histogram (*V(D)*). *OED *values are independent of the initial slope *β *and the modifying function *μ *and are thus keeping the necessary variables and the corresponding uncertainties at a minimum.

It should be noted here that for highly inhomogeneous dose distributions, cancer risk is proportional to average dose only for a linear dose-response relationship. For any other dose-response relationship, cancer risk is proportional to *OED*.

### Dose-response models for carcinoma induction

Several different dose-response relationships for carcinoma induction are considered here. The first is a linear response over the whole dose range:(6)

The second is a recently developed mechanistic model which accounts for cell killing and fractionation effects and is for carcinoma induction of the form [21]:(7)

where it assumed that the tissue is irradiated with a fractionated treatment schedule of equal dose fractions *d *up to a dose *D*. The number of cells is reduced by cell killing which is proportional to *α' *and is defined using the linear quadratic model(8)

where *D*_*T *_and *d*_*T *_is the prescribed dose to the target volume with the corresponding fractionation dose, respectively. For analyzing the Hodgkin data from Dores *et al. *[22] we used for *D*_*T *_= 40 Gy and for *d*_*T *_= 2 Gy. The repopulation/repair parameter *R *characterizes the repopulation/repair-ability of the tissue between two dose fractions and is 0 if no and 1 if full repopulation/repair occurs. It is assumed here an *α/β *= 3 Gy for all tissues, since analysis of breast cancer data has shown that the dose-response model is robust with variations in *α/β *[23].

Since a dose-response model as described by Eq. 7 is based on various assumptions and thus related to uncertainties it was decided to include two limiting cases. The first one, commonly named bell-shaped dose-response curve, is defined by completely neglecting any repopulation/repair effect and thus fractionation and is derived by taking Eq. 7 in the limit of *R = 0*:(9)

Although the case *R = 0 *represents an acute dose exposure, repopulation/repair effects are certainly important. However, any repopulated cell is not irradiated (as long as the time scale of irradiation is small) and thus, in the context of carcinogenesis, repopulation/repair effects are in this case irrelevant.

The second limiting case is a dose-response relationship in case of full repopulation/repair, and is derived by taking Eq. 7 in the limit of *R = 1*:(10)

Organ equivalent dose for the dose-response curves defined by Eqs. 6,7,9 and 10 become in the limit of small dose:(11)

Hence OED is, in the case of a homogenous distribution of small dose, average absorbed organ dose D;^-^. Thus in the limit of small dose all proposed dose-response relationships approach the LNT model and the initial slope *β *can be obtained from the most recent data for solid cancer incidence. Here the data for a follow-up period from 1958 to 1998 was used from a publication of Preston *et al. *[1].

### Dose-response models for sarcoma induction

The excess risk of sarcomas observed from the study of the A-bomb survivors [1] is an order of magnitude smaller than for carcinomas. Data from radiotherapy patients indicate however that sarcoma induction at high dose is at a comparable magnitude than carcinoma induction. Therefore it is not appropriate to assume a pure linear dose-response relationship for sarcoma induction. A recently developed sarcoma induction model was used which accounts for cell killing and fractionation effects and is based on the assumption that stem cells remain quiescent until external stimuli like ionizing radiation trigger re-entry into the cell cycle. The corresponding mechanistic model which accounts also for cell killing and fractionation effects is of the form [21]:(12)

where is assumed that the tissue is irradiated with a fractionated treatment schedule of equal dose fractions *d *up to a dose *D *and the parameters have the same meaning than in Eq. 7. Since a dose-response model as described by Eq. 12 is based on various assumptions and thus related to uncertainties it was decided, similar to the carcinoma case, to study three cases. The first one is defined by looking at minimal repopulation/repair effects by using Eq. 12 with a fixed *R = 0.1*. The second one is defined by looking at intermediate repopulation/repair effects by using Eq. 12 with a fixed *R = 0.5*. The third case is a dose-response relationship in case of full repopulation/repair, and is derived by taking Eq. 12 in the limit of *R = 1*:(13)

Organ equivalent dose for the dose-response curves for sarcoma induction defined by Eqs. 12 and 13 become, in the limit of small dose:(14)

Sarcoma risk from a homogenous distribution of small dose is proportional to the cube of dose and thus results in a much lower cancer risk than expected from a linear model. This is consistent with the observations of the A-bomb survivors.

### Modeling of the Hodgkin's patients

Cancer risk is only proportional to average organ dose as long as the dose-response curve is linear. At high dose it could be that the dose-response relationship is non-linear and as a consequence, *OED *replaces average dose to quantify radiation induced cancer. In order to calculate *OED *in radiotherapy patients, information on the three-dimensional dose distribution is necessary. This information is usually not provided in epidemiological studies on second cancers after radiotherapy. However, in Hodgkin's patients the three-dimensional dose distribution can be reconstructed.

For this purpose data on secondary cancer incidence rates in various organs for Hodgkin's patients treated with radiation were included in this analysis. Data on Hodgkin's patients treated with radiation seem to be ideal for an attempted combination with the A-bomb data. These patients were treated at a relatively young age, with curative intent and hence secondary cancer incidence rates for various organs are known with a good degree of precision. Since the treatment of Hodgkin's disease with radiotherapy has been highly successful in the past, the treatment techniques have not been modified very much over the last 30 years. This can be verified, for example, by a comparison of the treatment planning techniques used from 1960 to 1970 [24] with those used from 1980 until 1990 [25]. Additionally, the therapy protocols do not differ very much between the institutions that apply this form of treatment. These factors make it possible to reconstruct a statistically averaged *OED *for each dose-response model *RED(D)*, which is characteristic for a large patient collective of Hodgkin's disease patients.

The overall risk of selected second malignancies of 32,591 Hodgkin's patients after radiotherapy has been quantified by Dores *et al. *[22]. They found, for all solid cancers after the application of radiotherapy as the only treatment, an excess absolute risk of 33.1 per 10,000 patients per year. The site-specific excess risks are listed in Table 3. The total number of person years in these studies was 92,039 with a mean patient age at diagnosis of 37 years. The mean follow-up time of the Hodgkin's patients was 8 years. The mean age at diagnosis (*agex *= 37) and the mean attained age (*agea *= 45) was then used with the temporal patterns of the atomic bomb data (Eq. 2) to obtain the site specific risks at *agex *= 30 and *agea *= 70 years for the Dores data (Table 3). For bladder cancer the temporal pattern could be determined only with a large error which results in a variation of the corresponding *EAR*^*org *^by more than one order of magnitude. Therefore it was decided to apply for bladder cancer the temporal pattern for all solid cancers.

**Table 3:** Observed excess absolute risk of site-specific radiation induced cancer from the study of Dores *et al. *[22].

Site	***EAR***^***org***^ *agex *= 37 and *agea *= 45	***EAR***^***org***^ *agex *= 30 and *agea *= 70
All solid	33.1	112.1
Female breast	10.5	28.8
Lung	9.7	62.0
Rectum	0.40	1.53
Colon	2.0	62.4
Mouth and pharynx	2.7	9.1
Esophagus	0.7	1.6
Stomach	1.5	3.5
Small Intestine	0.1	3.1
Liver	0.4	2.3
Cervix	1.6	5.4
Bladder	0.8	2.7
Skin	0.9	9.5
Brain and CNS	0.5	1.7
Thyroid	1.4	2.5
Salivary Gland	0.5	1.7
Bone	0.3	0.3
Soft tissue	1.0	0.9

Patients were primarily treated for Hodgkin's disease with radiotherapy. The data for *agex *= 30 and *agea *= 70 years were converted using the temporal patterns of the atomic bomb data (Eq. 2) with the coefficients listed in Table 2.

Typical treatment techniques for Hodgkin's disease radiotherapy were reconstructed in an Alderson Rando Phantom with a 200 ml breast attachment. Treatment planning was performed on the basis of the review by Hoppe [25] and the German Hodgkin disease study protocols http://www.ghsg.org. We used for treatment planning the Eclipse External Beam Planning system version 8.6 (Varian Oncology Systems, Palo Alto, CA) using the AAA-algorithm (version 8.6.14) with corrected dose distributions for head-, phantom- and collimator-scatter. Three different treatment plans were computed which included a mantle field, an inverted-Y field and a para-aortic field. All plans were calculated with 6 MV photons and consisted of two opposed fields. The technique for shaping large fields included divergent lead blocks. Treatment was performed at a distance of 100 cm (SSD). Anterior-posterior (ap/pa) opposed field treatment techniques were applied to insure dose homogeneity.

The dose-volume histograms of the organs and tissues (exclusive of bone and soft tissue) which are listed in Table 1 were converted into *OED *according to the dose-response relationships for carcinomas (Eqs. 6, 7, 9 and 10). A statistically averaged *OED *was then obtained by combining the *OED *from different plans with respect to the statistical weight of the involvement of the individual lymph nodes [26]. The same was executed with bone and soft tissue using the sarcoma dose-response relationships from Eqs. 12 and 13. Here it is assumed that radiation causes in bone and soft tissue exclusively sarcomas, in all other organs which are listed in Table 1 carcinomas.

### Combined fit of A-bomb survivor and Hodgkin's patients

Since the dose distribution in a Hodgkin's patient is highly inhomogenous and the dose-response relationships as described by Eqs. 7, 9, 10, 12 and 13 are non-linear, it is not appropriate to apply a straight forward fit to the data. An iterative fitting procedure needs to be used instead. For this purpose, as described in the previous section, the dose-volume histograms for the different organs of interest were converted into *OED *for given model parameters *α *and *R*. The initial slope *β *was taken from Table 1 for carcinoma induction and kept fix. For sarcoma dose-response curves the parameters *β *and *α *were varied and *R *was kept fix at 0.1, 0.5 and 1, respectively.

The fitted *EAR *values were compared to the original data. The *α*- and *R*-values, and *α*- and *β*-values were fitted iteratively by minimizing χ^2 ^for carcinoma and sarcoma induction, respectively(15)

where the sum is taken over all bins of the dose volume histogram of the specific organ. The coefficient of variation (CV) was calculated to estimate the quality of the fit:(16)

A fit was accepted as significant good when CV < 0.05.

The linear model from Eq. 6 was optimized by allowing a variation of the initial slope *β *in the 95% confidence interval of the A-bomb survivor data (Table 1).

The procedure described above was slightly varied to fit all solid cancers, since for all solid cancers combined statistically significant A-bomb data up to approximately 5 Gy are available. Thus the *α- *value for all solid cancers combined could be obtained using the A-bomb data and was fixed at 0.089 [18].

## Results

**Figure 1:**
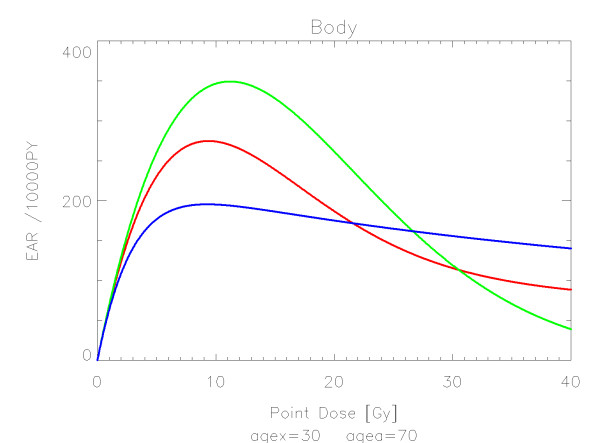
**Plot of excess absolute carcinoma risk for *all solid cancers *per 10,000 persons per year as a function of point dose in the organ**. The bell-shaped, plateau and full dose-response relationships are depicted by the green, blue and red line, respectively. The fits are presented for age at exposure of 30 years and attained age of 70 years.

The results of the parameter fits are listed in Table 4 for carcinoma induction and in Table 5 for sarcoma induction. Not all dose-response models could fit the data well (CV > 0.05). This was indicated by "nc" in the tables. The Figures show the fitted dose response models for the different organs and tissues. In Figures 1 (all solid), 2 (female breast), 3 (lung), 4 (colon), 5 (mouth and pharynx), 6 (stomach), 7 (small intestine), 8 (liver), 9 (cervix), 10 (bladder), 11 (skin), 12 (brain and CNS) and 13 (salivary glands) carcinoma induction is plotted using the linear model indicated by the black line, the full model marked by the red line, the model neglecting fractionation and thus repopulation with *R = 0 *(sometimes called a bell-shaped dose-response) labeled by the green line and finally the model describing full repopulation between dose fractions with *R = 1 *(sometimes called a plateau dose-response) marked by the blue line. Figures 14 (bone) and 15 (soft tissue) show sarcoma induction for the model with low repopulation effects and *R = 0.1 *labeled by the green line, with intermediate repopulation effects and *R = 0.5 *labeled by the red line and finally the model describing full repopulation between dose fractions with *R = 1 *marked by the blue line.

**Table 4:** Results of the fits to the Hodgkin data for the different dose-response models for carcinoma induction.

Site	Linear (Eq.6)		Full model (Eq.7)			No fractionation (bell shape) R = 0 (Eq.9)		Full tissue recovery (plateau) R = 1 (Eq.10)	
	***β***^†^	CV	α*	*R*	*CV*	α*	*CV*	α*	CV
All solid	nc		0.089	0.17	6.4E-3	0.065	4.8E-3	0.317	8.7E-4
Female breast	nc		0.044	0.15	1.1E-5	0.041	8.6E-4	0.115	1.9E-3
Lung	nc		0.042	0.83	2.0E-5	0.022	1.2E-2	0.056	1.7E-3
Rectum	nc		nc		8.7E-1	nc	8.7E-1	nc	8.7E-1
Colon	7.2	1.8E-4	0.001	0.99	7.1E-3	0.001	2.5E-2	0.001	2.0E-4
Mouth and pharynx	nc		0.043	0.97	3.8E-4	0.017	2.0E-3	0.045	6.6E-3
Esophagus	excluded								
Stomach	nc		0.460	0.46	8.4E-6	0.111	4.7E-3	nc	1.4E0
Small Intestine	nc		0.591	0.09	3.0E-5	0.480	2.9E-5	nc	3.2E0
Liver	0.22	3.4E-3	0.323	0.29	2.6E-5	0.243	3.4E-5	0.798	4.5E-2
Cervix	1.9	5.4E-4	nc		6.2E-1	nc	6.2E-1	nc	6.2E-1
Bladder	nc		0.219	0.06	1.9E-5	0.213	4.1E-4	0.633	1.0E-4
Skin	1.1	2.5E-3	nc		5.8E-1	nc	5.9E-1	nc	5.8E-1
Brain and CNS	0.44	9.8E-3	0.018	0.93	1.3E-4	0.009	4.8E-3	0.021	4.2E-3
Thyroid	excluded								
Salivary Gland	nc		0.087	0.23	3.4E-5	0.059	4.0E-3	0.282	2.2E-4

The fitted variables *β*, *α *and *R *are listed for each organ and each model. In addition the coefficient of variation (CV) is given. A fit corresponding to a CV > 0.05 was denoted as not converging (nc).

* in Gy^-1^

^† ^in (10,000 PY Gy)^-1^

**Table 5:** Results of the fit to the Hodgkin data for the different dose-response models for sarcoma induction.

Site	Low repopulation R = 0.1 (Eq.10)			Intermediate repopulation R = 0.5 (Eq.10)			Full tissue recovery R = 1 (Eq.11)		
	***β***^†^	α*	CV	***β***^†^	α*	CV	***β***^†^	α*	CV
Bone	1.70	0.019	2.1E-4	0.20	0.067	1.1E-3	0.10	0.078	4.3E-3
Soft tissue	3.30	0.040	1.9E-6	0.60	0.060	1.7E-4	0.35	0.093	5.8E-4

The fitted variables *β *and *α *are listed for each organ and for three different values for *R *(0.1, 0.5 and 1.0). In addition the coefficient of variation (CV) is given.

* in Gy^-1^

^† ^in (10,000 PY Gy)^-1^

**Figure 2:**
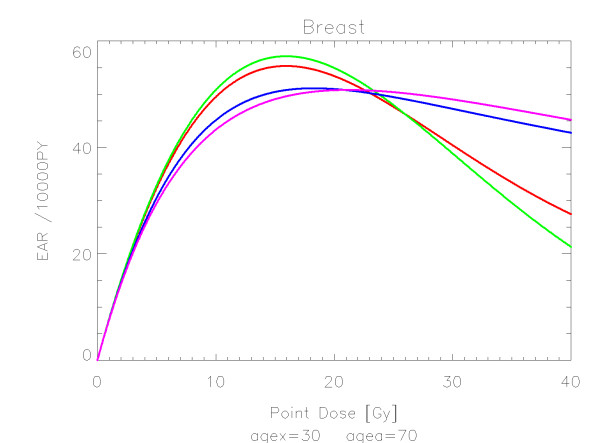
**Plot of excess absolute carcinoma risk for *female breast cancer *per 10,000 persons per year as a function of point dose in the organ**. The bell-shaped, plateau and full dose-response relationships are depicted by the green, blue and red line, respectively. The magenta curve represents the results from a fits to case control studies [23]. The fits are presented for age at exposure of 30 years and attained age of 70 years.

**Figure 3:**
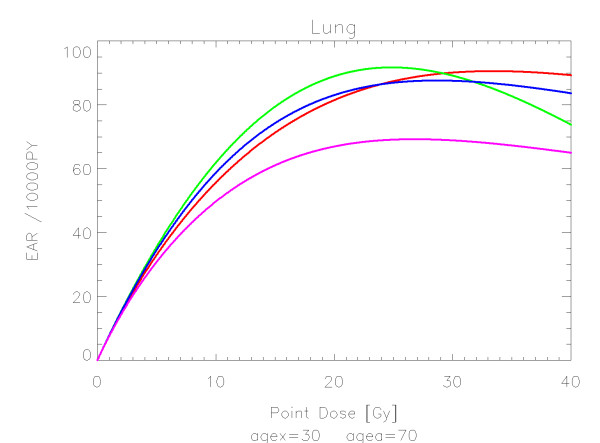
**Plot of excess absolute carcinoma risk for *lung cancer *per 10,000 persons per year as a function of point dose in the organ**. The bell-shaped, plateau and full dose-response relationships are depicted by the green, blue and red line, respectively. The magenta curve represents the results from a fits to case control studies [27]. The fits are presented for age at exposure of 30 years and attained age of 70 years.

**Figure 4:**
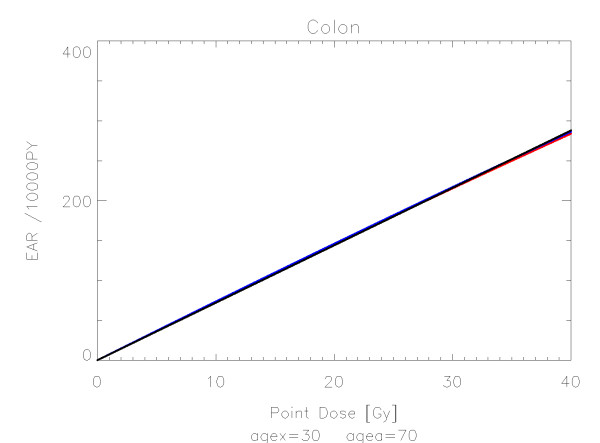
**Plot of excess absolute carcinoma risk for *colon cancer *per 10,000 persons per year as a function of point dose in the organ**. The linear, bell-shaped, plateau and full dose-response relationships are depicted by the black, green, blue and red line, respectively. The fits are presented for age at exposure of 30 years and attained age of 70 years.

**Figure 5:**
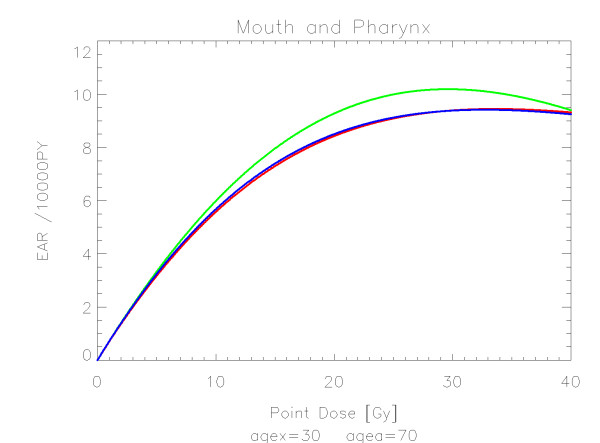
**Plot of excess absolute carcinoma risk for *cancers of the mouth and pharynx *per 10,000 persons per year as a function of point dose in the organ**. The bell-shaped, plateau and full dose-response relationships are depicted by the green, blue and red line, respectively. The fits are presented for age at exposure of 30 years and attained age of 70 years.

**Figure 6:**
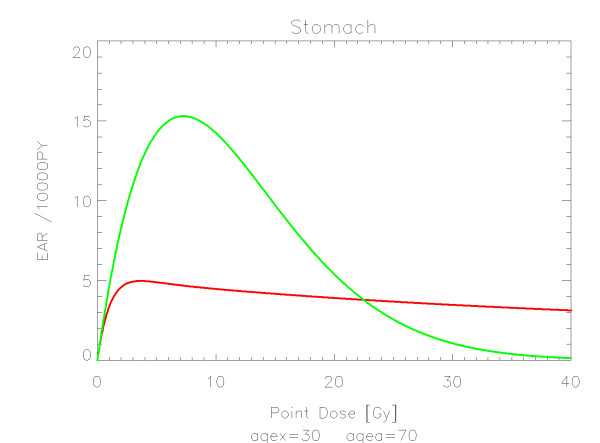
**Plot of excess absolute carcinoma risk for *stomach cancer *per 10,000 persons per year as a function of point dose in the organ**. The bell-shaped and full dose-response relationships are depicted by the green and red line, respectively. The fits are presented for age at exposure of 30 years and attained age of 70 years.

**Figure 7:**
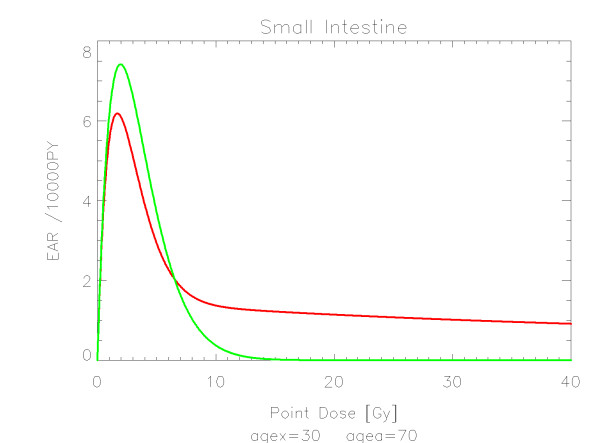
**Plot of excess absolute carcinoma risk for *cancer of the small intestine *per 10,000 persons per year as a function of point dose in the organ**. The bell-shaped and full dose-response relationships are depicted by the green and red line, respectively. The fits are presented for age at exposure of 30 years and attained age of 70 years.

**Figure 8:**
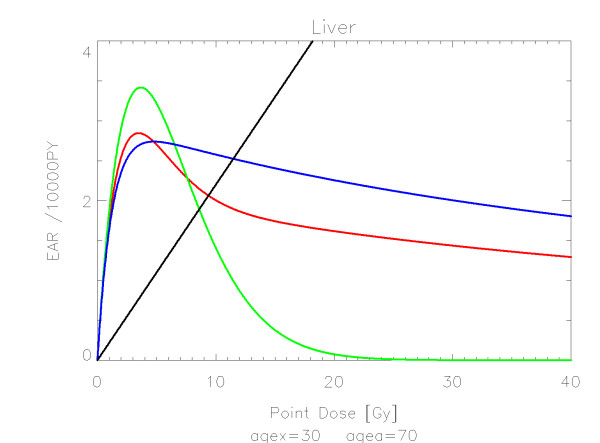
**Plot of excess absolute carcinoma risk for *liver cancer *per 10,000 persons per year as a function of point dose in the organ**. The linear, bell-shaped, plateau and full dose-response relationships are depicted by the black, green, blue and red line, respectively. The fits are presented for age at exposure of 30 years and attained age of 70 years.

**Figure 9:**
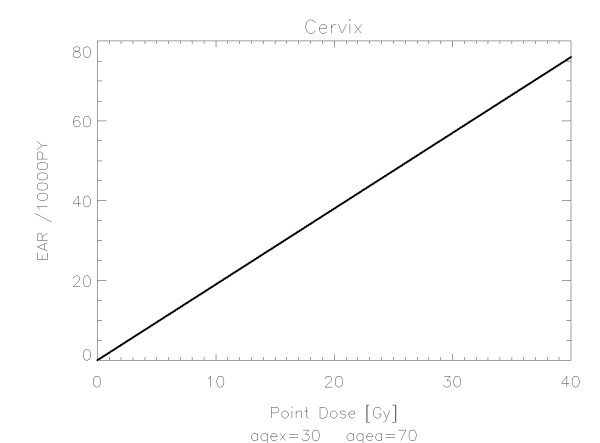
**Plot of excess absolute carcinoma risk for *cervix cancer *per 10,000 persons per year as a function of point dose in the organ**. The linear dose-response relationship is depicted by the black line. The fit is presented for age at exposure of 30 years and attained age of 70 years.

**Figure 10:**
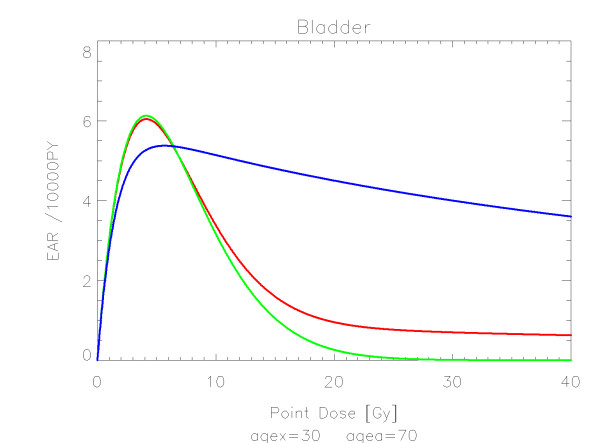
**Plot of excess absolute carcinoma risk for *bladder cancer *per 10,000 persons per year as a function of point dose in the organ**. The bell-shaped, plateau and full dose-response relationships are depicted by the green, blue and red line, respectively. The fits are presented for age at exposure of 30 years and attained age of 70 years.

**Figure 11:**
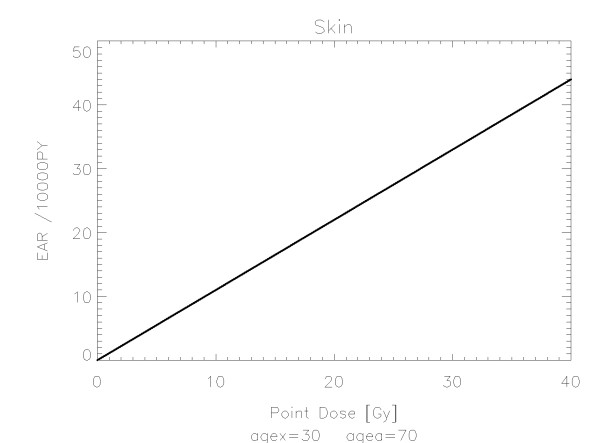
**Plot of excess absolute carcinoma risk for *skin cancer *per 10,000 persons per year as a function of point dose in the organ**. The linear dose-response relationship is depicted by the black line. The fit is presented for age at exposure of 30 years and attained age of 70 years.

**Figure 12:**
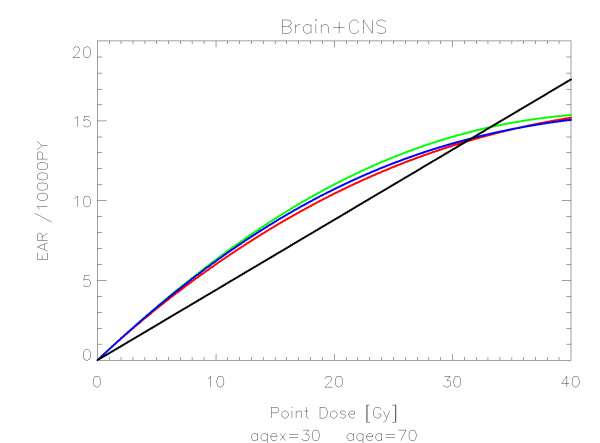
**Plot of excess absolute carcinoma risk for *cancer of the brain and CNS *per 10,000 persons per year as a function of point dose in the organ**. The linear, bell-shaped, plateau and full dose-response relationships are depicted by the black, green, blue and red line, respectively. The fits are presented for age at exposure of 30 years and attained age of 70 years.

**Figure 13:**
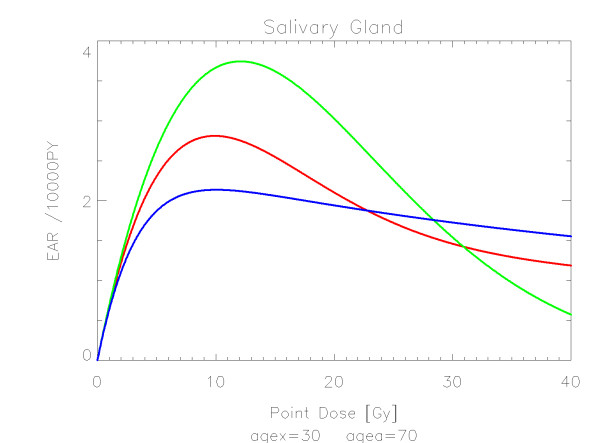
**Plot of excess absolute carcinoma risk for *cancer of the salivary glands *per 10,000 persons per year as a function of point dose in the organ**. The bell-shaped, plateau and full dose-response relationships are depicted by the green, blue and red line, respectively. The fits are presented for age at exposure of 30 years and attained age of 70 years.

**Figure 14:**
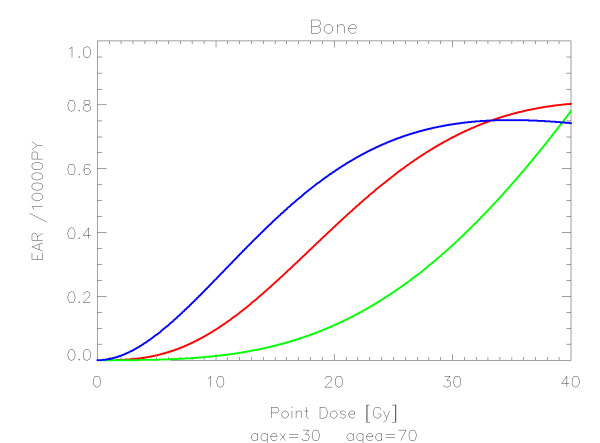
**Plot of excess absolute risk for *sarcoma incidence in bone *per 10,000 persons per year as a function of point dose in the organ**. The dose-response relationships representing low, intermediate, and full repopulation/repair are depicted by the green, red and blue line, respectively. The fits are presented for age at exposure of 30 years and attained age of 70 years.

**Figure 15:**
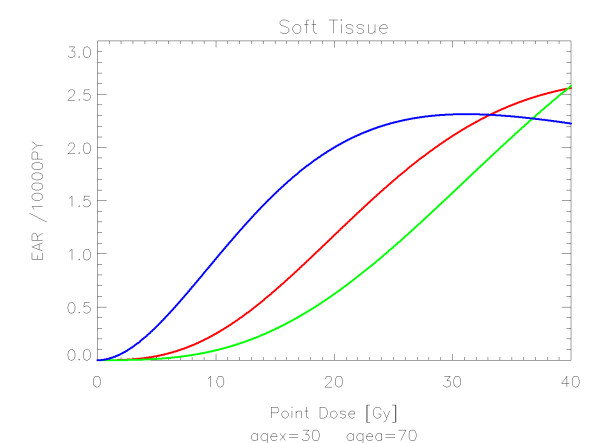
**Plot of excess absolute risk for *sarcoma incidence in soft tissue *per 10,000 persons per year as a function of point dose in the organ**. The dose-response relationships representing low, intermediate, and full repopulation/repair are depicted by the green, red and blue line, respectively. The fits are presented for age at exposure of 30 years and attained age of 70 years.

All dose-response models are plotted for a age at diagnosis of 30 and an attained age of 70 years, but they can easily converted to other ages by using the temporal patterns described by Eq. 2 with the parameters listed in Table 1.

From the analysis excluded were Esophagus and Thyroid, since theses organs were covered by a limited dose range of 30-55 Gy and 44-46 Gy, respectively.

## Discussion

Figure 1 shows the dose-response models fitted to the whole body structure (the complete Alderson phantom). The initial slope *β *of the A-bomb survivor data is that for all solid tumors. The linear model was not converging, all other models could be fitted. There is strong variation for intermediate dose levels around 10 Gy. It should be noted here that for inhomogenous dose distributions a dose response relationship for the whole body should be used with extreme care, as two completely different distributions of dose in the organs could result in the same *OED *for the whole body. The dose-response relationships for the whole body obtained in this report should be therefore used only for Hodgkin's patients treated with mantle fields. In contrast the dose-response relationships for single organs can be used generally for analyzing any dose distribution.

The quality of the applied fits shows that the linear model fits best colon, cervix and skin. All other organs are best fitted by the full model indicating that the repopulation/repair ability of tissue is neither 0 nor 100% but somewhere in between. It seems that for most organs at large doses the dose-response relationship is flattening or decreasing.

It should be noted that Mouth and Pharynx was covered by a limited dose range from 16-45 Gy. Thus the resulting dose-response relationship for dose levels outside that range should be used with care. For rectum none of the dose response models could predict the Hodgkin data. The linear model fitted colon, liver, cervix, skin and Brain/CNS. The model with full repopulation/repair did not fit rectum, cervix and skin.

Bone and soft tissue sarcoma were fitted by all the models well. In the low dose range beyond 1 Gy sarcoma risk is negligible. For increasing dose sarcoma risk increases rapidly and reaches a plateau at around 30 Gy. This is in agreement with observations which demonstrate small sarcoma risk at low dose from the A-bomb survivors and significant sarcoma risk in the high dose regions of radiotherapy patients.

The results of this study can be compared to EAR-modeling based on case control studies. In two recent publications excess absolute risk of breast and lung cancer was fitted to the model including fractionation [23,27]. For breast cancer the obtained model parameters where *α *= 0.067 Gy^-1 ^and *R *= 0.62 and for lung *α *= 0.061 Gy^-1 ^and *R *= 0.84. The corresponding dose-response curves are plotted in Figures 2 and 3 as the magenta lines for comparison with the results obtained in this study. If it is considered that the dose-response relationships were derived from two completely different data sets with two different methods the agreement is satisfying.

The epidemiological data from the Atomic-bomb survivors and the Hodgkin's patients are associated with large errors as discussed below. Nevertheless some basic conclusion can be tentatively drawn from the analysis presented here.

Increased risks of solid cancers after Hodgkin's disease have been generally attributed to radiotherapy. An important question is whether chemotherapy for Hodgkin's disease also adds to the solid cancer risk, and if so, at which sites. If chemotherapy indeed affects induction of solid tumors, one would expect that patients receiving combined modality treatment would have a greater relative risk than patients treated solely with radiotherapy. In several studies, no increased risk of solid cancers overall was observed after the application of chemotherapy alone. Dores *et al. *[22] calculated both the risk after radiotherapy alone and the solid cancer risk after combined modality therapy and found an excess absolute risk of 39 and 43 per 10,000 patients per year, respectively. As a consequence, the difference in risk between combined modality treatment and radiotherapy alone (4 per 10,000 patients per year) can be tentatively attributed to either chemotherapy or a genetic susceptibility of the Hodgkin patient population with regard to cancer or both. The risk difference accounts approximately for 10% of all solid cancers and can be regarded as not substantial when compared to other errors involved for risk estimation and is also not statistically significant (see Table 1).

It is well known that genetic susceptibility underlies Hodgkin's disease [28]. It is not clear whether this genetic susceptibility would also affect the development of other cancers. There is the possibility of a cancer diathesis, the prospect that, for some reasons related to genetic makeup, a person who developed one cancer has an inherently increased risk of developing another. However, such cancer susceptibility would result in a minimal excess cancer incidence compared to the incidence of radiation related tumors, since such an excess cancer incidence of solid tumors should also be seen in Hodgkin's patients after treatment with chemotherapy alone. However, there is no statistically significant increase for all solid tumors combined. Therefore, such an effect will be hidden in the 95% confidence interval of the observed cancer incidence after chemotherapy.

In this work *EAR *has been used to quantify radiation-induced cancer. *EAR *is used here, since the risk calculations of the Hodgkin's cohort are based on extremely inhomogenous dose distributions. It is assumed that the total absolute risk in an organ is the volume weighted sum of the risks of the partial volumes which are irradiated homogenously. Currently there is no available method for obtaining analogous organ risks using *ERR *without modeling the underlying baseline risk. Shuryak *et al. *[16,17] recently published a model including the description of typical background carcinogenesis in addition to radiation induced cancer. They could thus obtain a microscopic *ERR *model. The advantage of their model in comparison to our approach is that they could determine directly *ERR *the disadvantage is a larger number of adjustable parameters (three more parameters) which must be introduced to model background cancer risk.

The A-bomb survivor data used in this work were taken from a recent report from Preston *et al. *[1]. Preston determined the initial slope of the dose-response relationship by using an RBE of 10 for the neutrons. Recent research by Sasaki *et al. *however indicated that the neutron RBE might by larger and varying with dose [29]. It could be important to determine site specific cancer induction also for a dose varying RBE similar to the work which was done for all solid cancers combined [18].

## Conclusions

A comparison of dose distributions in humans, for example in radiotherapy treatment planning, with regard to cancer incidence or mortality can be performed by computing *OED*, which can be based on any dose-response relationship. In this work *OED *for various organs was calculated for a linear, a bell-shaped, a plateau and a mixture between a bell-shaped and plateau dose-response relationship for typical treatment plans of Hodgkin's disease patients. The model parameters (*α *and *R*) were obtained by a fit of the dose-response relationships to these *OED *data and to the A-bomb survivors. For any three-dimensional inhomogenous dose distribution, cancer risk can be compared by computing *OED *using the coefficients obtained in this work.

For absolute risk estimates, *EAR*^*org *^can be determined by taking additionally the initial slope *β *from Table 1 and multiplying it with the population-dependent modifying function using the coefficients of Table 1. However, absolute risk estimates must be viewed with care, since the errors involved are large.

## Competing interests

The authors state that there is no conflict of interest for the authors or the author's institution and that they have no financial or personal relationships that inappropriately influence their actions. They have no dual commitments, competing interests, competing loyalties, employment, consultancies, stock ownership, honoraria, or paid expert testimony.

## Authors' contributions

US designed this study, performed the modeling, and drafted the manuscript. MS and JR performed the treatment planning and the dose reconstruction for the risk predictions. All authors read and approved the final manuscript.

## References

[B1] Preston DL Ron E Tokuoka S Funamoto S Nishi N Soda M Mabuchi K Kodama K Solid cancer incidence in atomic bomb survivors: 1958-1998 Radiat Res 2007 168 1 1 64 10.1667/RR0763.1 17722996

[B2] Preston DL Pierce DA Shimizu Y Cullings HM Fujita S Funamoto S Kodama K Effects of recent changes in Atomic bomb survivor dosimetry on cancer mortality risk estimated Radiat Res 2004 162 377 389 10.1667/RR3232 15447045

[B3] Walsh L Rühm W Kellerer AM Cancer risk estimates for X-rays with regard to organ specific doses, part I: All solid cancers combined Radiat Environ Biophys 2004 43 145 151 10.1007/s00411-004-0248-5 15309386

[B4] Walsh L Rühm W Kellerer AM Cancer risk estimates for γ-rays with regard to organ specific doses, part II: Site specific solid cancers Radiat Environ Biophys 2004 43 225 231 10.1007/s00411-004-0263-6 15645312

[B5] Lindsay KA Wheldon EG Deehan C Wheldon TE Radiation carcinogenesis modelling for risk of treatment-related second tumours following radiotherapy Br J Radiol 2001 74 882 529 36 11459732 10.1259/bjr.74.882.740529

[B6] Hall EJ Wuu CS Radiation-induced second cancers: the impact of 3D-CRT and IMRT Int J Radiat Oncol Biol Phys 2003 56 1 83 8 10.1016/S0360-3016(03)00073-7 12694826

[B7] Davis RH Production and killing of second cancer precursor cells in radiation therapy: in regard to Hall and Wuu (Int J Radiat Oncol Biol Phys 2003;56:83-88) Int J Radiat Oncol Biol Phys 2004 59 3 916 and author reply *Int J Radiat Oncol Biol Phys. *2005 **Jan 1;61(1)**:312-3 10.1016/j.ijrobp.2003.09.076 15183501

[B8] Schneider U Besserer J Mack A Hypofractionated radiotherapy has the potential for second cancer reduction Theor Biol Med Model 2010 7 4 10.1186/1742-4682-7-4 20149259 PMC2829001

[B9] Dasu A Toma-Dasu I Dose-effect models for risk-relationship to cell survival parameters Acta Oncol 2005 44 8 829 35 10.1080/02841860500401159 16332590

[B10] Dasu A Toma-Dasu I Olofsson J Karlsson M The use of risk estimation models for the induction of secondary cancers following radiotherapy Acta Oncol 2005 44 4 339 47 10.1080/02841860510029833 16120542

[B11] Schneider U Zwahlen D Ross D Kaser-Hotz B Estimation of radiation-induced cancer from three-dimensional dose distributions: Concept of organ equivalent dose Int J Radiat Oncol Biol Phys 2005 61 5 1510 5 10.1016/j.ijrobp.2004.12.040 15817357

[B12] Schneider U Kaser-Hotz B A simple dose-response relationship for modeling secondary cancer incidence after radiotherapy Z Med Phys 2005 15 1 31 7 15830782 10.1078/0939-3889-00242

[B13] Sachs RK Brenner DJ Solid tumor risks after high doses of ionizing radiation Proc Natl Acad Sci USA 2005 102 37 13040 5 10.1073/pnas.0506648102 16150705 PMC1199000

[B14] Schneider U Kaser-Hotz B Radiation risk estimates after radiotherapy: application of the organ equivalent dose concept to plateau dose-response relationships Radiat Environ Biophys 2005 44 3 235 9 10.1007/s00411-005-0016-1 16273381

[B15] Pfaffenberger A Schneider U Poppe B Oelfke U Phenomenological modelling of second cancer incidence for radiation treatment planning Z Med Phys 2009 19 4 236 50 19962082 10.1016/j.zemedi.2009.08.002

[B16] Shuryak I Hahnfeldt P Hlatky L Sachs RK Brenner DJ A new view of radiation-induced cancer: integrating short- and long-term processes. Part I: approach Radiat Environ Biophys 2009 48 3 263 74 10.1007/s00411-009-0230-3 19536557 PMC2714893

[B17] Shuryak I Hahnfeldt P Hlatky L Sachs RK Brenner DJ A new view of radiation-induced cancer: integrating short- and long-term processes. Part II: second cancer risk estimation Radiat Environ Biophys 2009 48 3 275 86 10.1007/s00411-009-0231-2 19499238 PMC2714894

[B18] Schneider U Walsh L Cancer risk estimates from the combined Japanese A-bomb and Hodgkin cohorts for doses relevant to radiotherapy Radiat Environ Biophys 2008 47 2 253 63 10.1007/s00411-007-0151-y 18157543

[B19] The 2007 Recommendations of the International Commission on Radiological Protection. ICRP publication 103 Ann ICRP 2007 37 2-4 1 332 18082557 10.1016/j.icrp.2007.10.003

[B20] UNSCEAR United Nations Scientific Committee on the Effects of Atomic Radiation (2006) Effects of ionizing radiation UNSCEAR 2006 Report to the General Assembly, with Scientific Annex United Nations, New York

[B21] Schneider U Mechanistic model of radiation-induced cancer after fractionated radiotherapy using the linear-quadratic formula Med Phys 2009 36 4 1138 43 10.1118/1.3089792 19472619

[B22] Dores GM Metayer C Curtis RE Lynch CF Clarke EA Glimelius B Storm H Pukkala E van Leeuwen FE Holowaty EJ Andersson M Wiklund T Joensuu T van't Veer MB Stovall M Gospodarowicz M Travis LB Second malignant neoplasms among long-term survivors of Hodgkin's disease: a population-based evaluation over 25 years J Clin Oncol 2002 20 16 3484 94 10.1200/JCO.2002.09.038 12177110

[B23] Schneider U Sumila M Robotka J Gruber G Mack A Besserer J Dose-response relationship for breast cancer induction at radiotherapy dose Radiat Oncol 2011 6 1 67 10.1186/1748-717X-6-67 21651799 PMC3127785

[B24] Carmel RJ Kaplan HS Mantle irradiation in Hodgkin's disease. An analysis of technique, tumor eradication, and complications Cancer 1976 37 6 2813 25 10.1002/1097-0142(197606)37:6<2813::AID-CNCR2820370637>3.0.CO;2-S 949701

[B25] Hoppe RT Radiation therapy in the management of Hodgkin's disease Semin Oncol 1990 6 704 15 2251517

[B26] Mauch PM Kalish LA Kadin M Coleman CN Osteen R Hellman S Patterns of presentation of Hodgkin disease. Implications for etiology and pathogenesis Cancer 1993 71 6 2062 71 10.1002/1097-0142(19930315)71:6<2062::AID-CNCR2820710622>3.0.CO;2-0 8443755

[B27] Schneider U Stipper A Besserer J Dose-response relationship for lung cancer induction at radiotherapy dose Z Med Phys 2010 20 3 206 14 20832008 10.1016/j.zemedi.2010.03.008

[B28] Mack TM Cozen W Shibata DK Weiss LM Nathwani BN Hernandez AM Taylor CR Hamilton AS Deapen DM Rappaport EB Concordance for Hodgkin's disease in identical twins suggesting genetic susceptibility to the young-adult form of the disease N Engl J Med 1995 332 7 413 8 10.1056/NEJM199502163320701 7824015

[B29] Sasaki MS Nomura T Ejima Y Utsumi H Endo S Saito I Itoh T Hoshi M Experimental derivation of relative biological effectiveness of A-bomb neutrons in Hiroshima and Nagasaki and implications for risk assessment Radiat Res 2008 170 1 101 17 10.1667/RR1249.1 18582156

